# Study on Early-Age Capillary Pressure and Plastic Shrinkage Properties of High-Volume Fly Ash Concrete

**DOI:** 10.3390/ma18081884

**Published:** 2025-04-21

**Authors:** Jintao Liu, Xinyang Yu, Shaojiang Wang, Jie Yang, Qianni Cai

**Affiliations:** 1 College of Civil Engineering, Zhejiang University of Technology, Hangzhou 310023, China; jtliu@zjut.edu.cn (J.L.); 2112106079@zjut.edu.cn (X.Y.); 2112006098@zjut.edu.cn (Q.C.); 2 Key Laboratory of Civil Engineering Structures & Disaster Prevention and Mitigation Technology of Zhejiang Province, Zhejiang University of Technology, Hangzhou 310023, China; 3 China Institute of Water Resources and Hydropower Research, Beijing 100038, China; 4 FangYuan New Materials Co., Ltd., Taizhou 318000, China

**Keywords:** high-volume fly ash concrete (HVFAC), early age, capillary pressure, plastic shrinkage

## Abstract

There is a lack of research on the early plastic deformation and capillary pressure of high-volume fly ash concrete (HVFAC) under varying ambient temperatures. This study aims to investigate the effects of water–binder ratio, fly ash admixture, and ambient temperature on the air entry time *T*, capillary pressure, and plastic shrinkage of HVFAC. Nine different fly ash concrete materials were designed and analyzed to determine the early plastic deformation and capillary pressure of HVFAC under different ambient temperatures. The dosage of different superplasticizers was adjusted to ensure a slump of 180 mm for all the HVFAC mixtures. The results showed that at 20 °C, *T* increases with the increase in the water–binder ratio and fly ash admixture, while the effect of *T* is negligible at 35 °C. The plastic shrinkage of HVFAC increases significantly with the increase in curing temperature, and there is a linear correlation between the air entry time *T* and the plastic shrinkage value at this time. At low water–binder ratios, the capillary pressure threshold *P_a_* increases with increasing curing temperature, while at high water–binder ratios, there is no significant trend observed for *P_a_*. The findings of the study can provide a theoretical basis for preventing plastic cracking of concrete and optimizing early curing methods.

## 1. Introduction

High-volume fly ash concrete (HVFAC) is a type of concrete that contains a significant amount of fly ash [1]. The spherical morphology of fly ash and the effects of volcanic ash and micro-aggregates can effectively reduce the early hydration and shrinkage of concrete [2]. Due to its environmental benefits and economic advantages, HVFAC has been widely used in large-volume concrete projects such as bridges, roads, and water conservancies in recent years [3,4,5,6,7,8].

Crack resistance is a crucial factor that affects the durability of concrete structures. Early cracking in concrete can lead to a larger number of cracks and a reduced expected service life of the structure. Typically, early-age cracking in concrete occurs primarily due to plastic shrinkage caused by water evaporation [9,10,11,12,13,14].

Meanwhile, concrete may also crack at later stages due to factors such as thermal deformation [15], autogenous shrinkage [16,17], and drying shrinkage [18]. The high volume of fly ash in concrete delays the hydration reaction process, leading to more free water remaining in the mixture. As a result, the water evaporation rate increases, causing greater early-age plastic shrinkage in HVFAC under drying conditions [19]. Moreover, the newly mixed HVFAC is in an elastic–plastic state in the early stage, and early plastic cracking may occur when concrete shrinkage is restrained by the coarse aggregate and reinforcement [20].

The physical process of plastic shrinkage in concrete involves the accumulation of capillary pressure within the liquid phase of the concrete. This capillary pressure exerts forces on cement particles, leading to the plastic shrinkage of concrete [10]. Jamali et al. [21] employed a high-capacity tensiometer to test the capillary pressure of early-age concrete for the first time and proposed a new four-stage model. Ghourchian et al. [9] proposed a poromechanics model for the plastic shrinkage of cementitious materials and found that capillary pressure prior to air ingress makes the bulk modulus larger and accelerates cracking. Similarly, Hu et al. [22] proposed a multiscale autogenous shrinkage prediction model based on capillary pressure. Thus, early-age capillary pressure monitoring in concrete serves as a critical indicator for evaluating shrinkage-induced cracking mechanisms [23]. During the growth of capillary pressure, air infiltrates the concrete matrix through the largest pore on the concrete surface, resulting in the formation of microcracks, and this time point is known as the air entry time (*T*). A higher *T* value indicates delayed microcrack formation and improved early crack resistance of concrete. Slowik et al. [24] clarified the relationship between *T* and the early plastic cracking of concrete. Simulation of Capillary Shrinkage has been found to effectively reduce the risk of plastic cracking in concrete.

Monitoring the evolution of capillary pressure in early-age concrete is crucial for predicting and analyzing crack formation [25]. If capillary pressure is kept to a low level and the air entry time is postponed through reasonable curing methods, the risk of early plastic cracking of the concrete will be significantly reduced. However, it is difficult to accurately determine air entry time by capillary pressure of concrete [26]. Some studies use concrete resistivity as an indicator of plastic cracking and local pore changes in concrete. McCarter et al. [27] found that the resistivity fluctuated at some point during the early age. The resistivity changed significantly during the period when the paste was gaining rigidity. Slowik et al. [24] found that air entry time (*T*) could be captured by resistivity tests, exhibiting a sudden change in the derivative of resistivity.

Concrete structures in practical engineering are often directly exposed to the environment at an early age, including curing conditions such as high temperature and drying conditions. The direct measurement of capillary pressure is difficult [21]; the existing measurements of capillary pressure mainly focus on ordinary concrete [28,29,30,31,32]. The early hydration process of HVFAC is slow, resulting in more pronounced early plastic shrinkage. Hence, this study aims to investigate the effects of water–binder ratio (0.4, 0.5, and 0.6), fly ash content (0, 30%, and 60%), and ambient temperatures (20 °C and 35 °C) on the capillary pressure and plastic shrinkage of HVFAC. Finally, the early-age crack resistance of HVFAC was evaluated and discussed by analyzing the air entry time (*T*), capillary pressure, and plastic shrinkage of each HVFAC. The air entry time (*T*) of each HVFAC was determined by the sudden change in the derivative of resistivity. The findings obtained in this research could offer a theoretical basis for preventing the plastic cracking of concrete and optimizing the early curing methods.

## 2. Materials and Methods

### 2.1. Raw Materials and Mix Proportion

The cement used in this study was P·I cement of 42.5 strength grade produced by China United Cement Group Co., Ltd., Beijing, China. The specific surface area of cement was 355 m^2^/kg and the apparent density was 3.16 g/cm^3^. Chemical analyses of SiO_2_, Al_2_O_3_, Fe_2_O_3_, CaO, MgO, and SO_3_ were performed by XRF with the use of a Wavelength-Dispersive XRF spectrometer (ThermoFisher Scientific Co., Ltd., Waltham, MA, USA, ADVANT’X 4200). Particle size distributions of the materials were obtained by using Mastersizer 2000(Malvern Panalytical Co., Ltd., Malvern, UK). X-ray diffraction (XRD) patterns were recorded with a PANalytical X’Pert PRO (Malvern Panalytical Co., Ltd., Malvern, UK) powder diffractometer using Cu Kα radiation (λl = 0.1541 nm). The patterns were collected with a 2θ range from 10° to 80° at a step size of 0.026°.

In this study, river sand and crushed limestone were used as fine and coarse aggregates, respectively. The river sand had an apparent density of 2610 kg/m^3^ and a fineness modulus of 2.6, while the apparent density of gravel was 2670 kg/m^3^. The particle grading of fine and coarse aggregates is shown in Table 1 and Table 2. Both of them met the quality requirements specified in the Standard JGJ 52-2006 [33]. The water used in the study was Hangzhou municipal tap water, and the admixture used was MELFLUX 4930F polycarboxylic acid superplasticizer with the 30% water-reducing rate produced by BASF, Rhineland-Palatinate, Germany. The PH value of the superplasticizer is 7.4.

The concrete mix proportion of each group is presented in Table 3. The cement in the concrete was replaced by fly ash at equal masses of 30% and 60%, respectively, and the consistency of the concrete slump was controlled by adjusting the content of the water-reducing agent. The dosage of different superplasticizers was adjusted to ensure a slump of 180 mm for all the HVFAC mixtures according to the Chinese standard “Technical Specification for Concrete Pumping Construction” (JGJ/T 10-2011) [34]. The 28 d compressive strength under standard curing conditions (20 ± 2 °C, 95% RH) was also listed in Table 3. Due to the different densities of fly ash and cement, when replacing cement with fly ash on an equal mass basis, the unit mass of the mixture will change. Therefore, the mix proportions in Table 3 are scaled to meet the actual requirements.

### 2.2. Test Methods

#### 2.2.1. Electrical Resistivity

In this study, concrete resistivity was tested using the four-electrode method. The specimens were 100 mm × 100 mm × 400 mm in dimension. Four parallel copper meshes with a distance of 80 mm were embedded in the concrete mold. Two outer copper meshes were used as the current electrodes, and two middle copper meshes were used as the voltage electrodes. After the concrete was finished casting, a PLD-6005 DC power supply (Henghui Co., Ltd., Shengzhen, China) was used to provide a constant 10 mA current, and a TDS-530 data acquisition instrument (Tokyo MeasuringInstruments Lab Co., Ltd., Tokyo, Japan) was used to collect the voltage value of the middle copper meshes. The resistivity of the specimen was calculated with the equation [35] as follows:

(1) $$\rho =\frac{RS}{L}=\frac{U\cdot S}{I\cdot L}$$

where *ρ* is the resistivity of the specimen (Ω·m); *U* is the voltage value of the specimen (V); *I* is the current value passing through the specimen (A); *S* is the cross-sectional area (100 mm × 100 mm) of the specimen; and *L* is the length of two middle copper meshes, taken as 80 mm.

#### 2.2.2. Capillary Pressure

**Figure 1:**
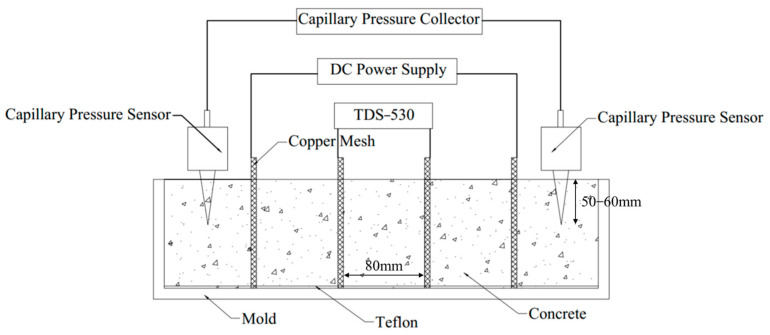
Resistivity and capillary pressure tests of concrete.

Capillary pressure was measured using a system provided by Chiao Broad Link (710) (CHIAO BROAD LINK(Beijing)Equipment Co, Ltd., Beijing, China) consisting of a data acquisition system, pressure sensors, cone-shaped tubes, and shielded lines. Capillary pressure tests and resistivity tests were performed on the same specimen at the same time. After the concrete was finished casting, a wooden rod with a similar thickness to the cone-shaped tube was inserted 60–70 mm into the concrete surface to displace the coarse aggregate in that position. As shown in Figure 1, the cone-shaped tube was then inserted and fixed, penetrating 50–60 mm into the concrete surface. Before the start of the test, we needed to fill pure water in the conical tube, co, nnect the syringe and the conical tube to the opening, pump the syringe so that the negative pressure inside the conical tube was maintained, and after a period of time, knock the conical tube wall to discharge the air bubbles. We needed to repeat the above steps until there were no air bubbles in the tube, which was the value of the machine on the clearing of the zero before the start of the test. When the concrete surface was slightly dry and the cone-shaped tube could maintain itself at that depth, the fixation of the cone-shaped tube was loosened, allowing it to settle down together with the concrete. The cone-shaped tube was attached to the water in the concrete to form a system, and the pressure sensor could measure the capillary pressure inside the concrete.

#### 2.2.3. Plastic Shrinkage

The plastic shrinkage test was carried out with reference to GB/T 50082-2009 [36]. The test was carried a non-contact concrete shrinkage deformation device of CABR-NES-E type provided by Zhoushan Boyuan Technology Development Co., Ltd., Zhoushan, China. The size of the specimen was 100 mm × 100 mm × 400 mm. To reduce friction between the concrete specimen and mold, a Teflon plate was placed at the bottom of the mold, and 5 mm thick foam plates were placed on both sides of the end plates. After that, non-contact displacement sensors were fixed at both ends of the test mold.

Two parallel specimens were prepared for each group without film coating. The ambient humidity was controlled at 60 ± 5% RH, while the temperature was set at 20 ± 2 °C and 35 ± 2 °C. The specimens under different curing temperatures were numbered; for example, A3-20 refers to the concrete of 0.4 water–binder ratio with 30% fly ash content cured at 20 °C.

#### 2.2.4. Heat of Hydration

The heat of hydration of the cementitious paste was measured using an eight-channel isothermal calorimeter (TAM AIR, USA/TA Instruments-Waters LLC Co., Ltd., New Castle, DE, USA). Each channel of the isothermal calorimeter contains two chambers, consisting of a sample cell and a reference cell. Circulating air was used as the medium in the constant temperature bath, and the system was adjusted to ensure temperature stability. The isothermal calorimetry measurements were performed at temperatures of 20 °C and 35 °C, with exothermic heat release data continuously recorded for a duration of 72 h. The heat of hydration measurements was systematically conducted on a total of 18 cementitious paste mixtures, taking into account variations in fly ash content (0%, 30%, and 60%), water–binder ratios (0.4, 0.5, and 0.6), and curing temperatures (20 °C and 35 °C).

## 3. Results

### 3.1. Raw Materials and the Heat of Hydration

**Figure 2:**
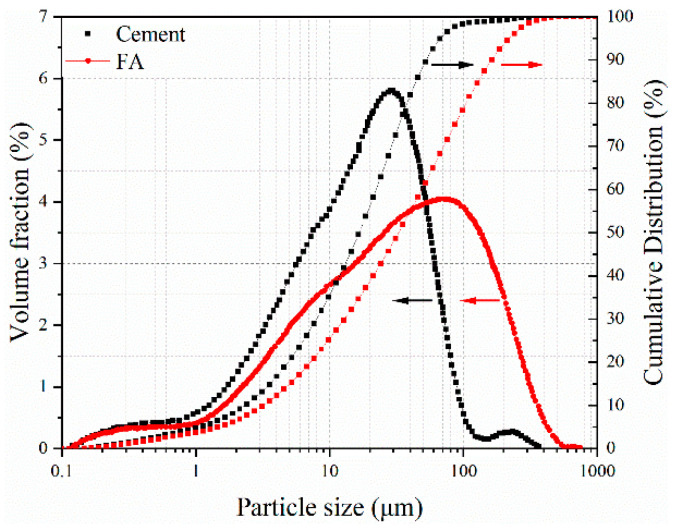
Particle size distribution curves of cement and fly ash.

**Figure 3:**
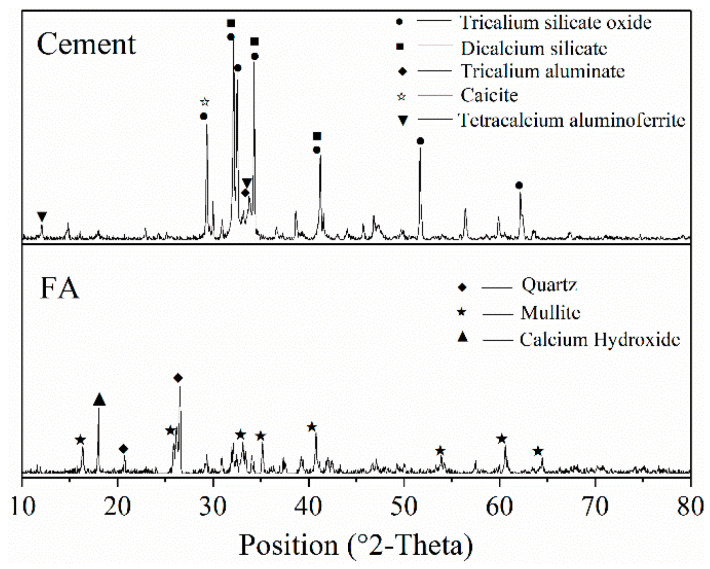
XRD pattern of cement and fly ash.

The grade of fly ash was Class II, with a water requirement ratio of 96.4% and an activity index of 73.0%. The X-ray fluorescence (XRF) test results for the chemical composition of the cement and fly ash are listed in Table 4. The particle size distributions of the cement and fly ash are shown in Figure 2. The result of XRD is shown in Figure 3.

**Figure 4:**
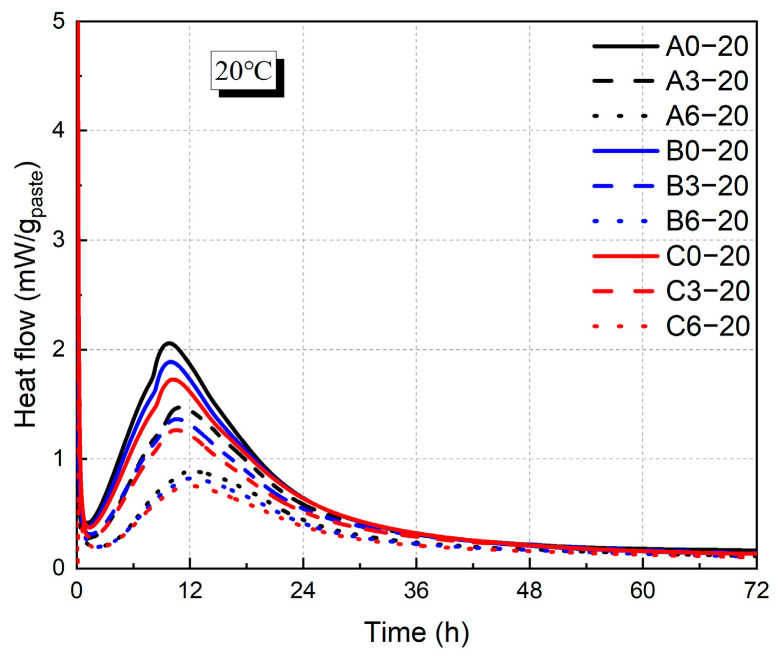
Heat flow of cement paste at 20 °C.

**Figure 5:**
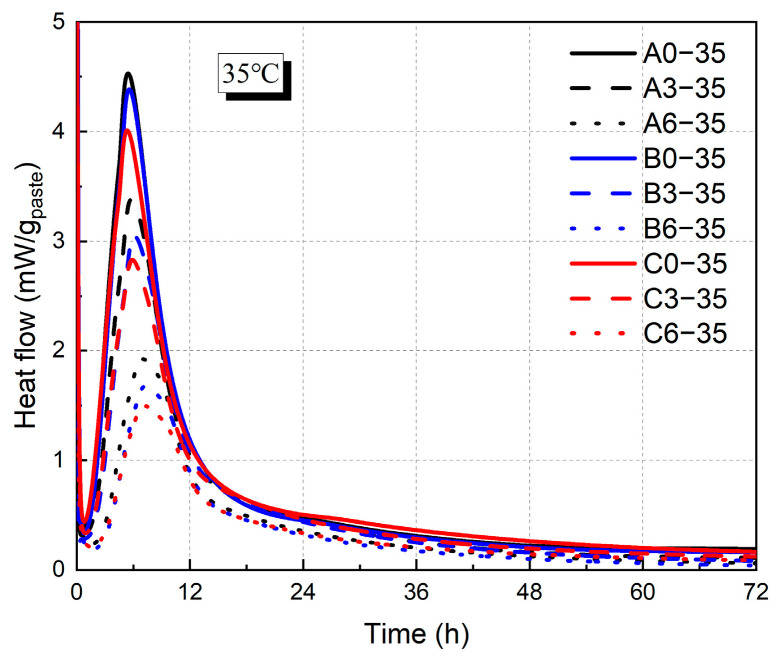
Heat flow of cement paste at 35 °C.

As shown in Figure 4 and Figure 5, the incorporation of fly ash decreased the heat release rate of hydration. At 20 °C, increasing the fly ash admixture from 0% to 60% resulted in reductions in hydration heat by 57%, 50%, and 53% for water–binder ratios of 0.3, 0.4, and 0.5, respectively. In contrast, variations in the water–binder ratio exhibited a relatively minor influence on hydration heat, with an increased water–binder ratio leading to a moderate reduction in the exothermic rate. At 20 °C, for cementitious matrices containing 0% fly ash, an increase in the water–binder ratio from 0.3 to 0.6 led to a 19% decrease in the peak heat of hydration. Moreover, this declining trend diminished with an increase in fly ash replacement, and at a 60% fly ash replacement, the peak hydration heat decreased by 14%. With higher temperatures, the exothermic rate was higher, and the time to reach the exothermic peak was advanced from 10 h to 4.5 h. For the group with a water–binder ratio of 0.3 and 0% fly ash content, the heat of hydration at 35 °C exhibited a pronounced increase of 114%.

### 3.2. Early Age of Capillary Pressure, Resistivity, and Plastic Shrinkage of Concrete

**Figure 6:**
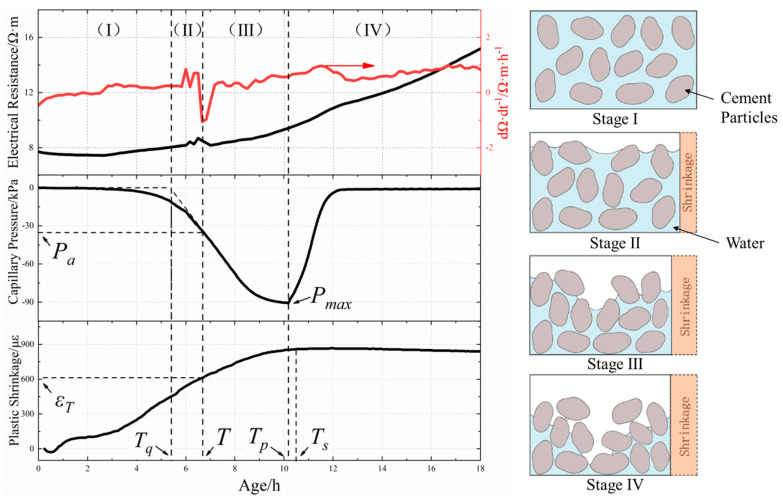
The development law of early-age resistivity, capillary pressure, and shrinkage and schematic diagram of concrete.

The development law of capillary pressure, resistivity, and plastic shrinkage of concrete is illustrated in Figure 6. When cementitious materials are mixed with water, they gradually dissolve a large number of charged ions, such as Ca^2+^, OH^−^, K^+^, and Na^+^. These charged ions generate directional motion and form current with the impact of an applied electric field. During the process of concrete hydration and water evaporation, the free water in the concrete matrix was continuously consumed, leading to a gradual decrease in the volume fraction of the liquid phase. Meanwhile, the C-S-H and AFt generated by the hydration reaction increased the volume fraction of the solid phase in the matrix and caused an upward trend in the resistivity curve. As the water was gradually consumed, the water film on the surface of the concrete matrix, which is formed by bleeding, decreased until it could no longer fully cover the entire surface. At this stage, air began to enter the matrix from different areas of the surface, which affected its resistivity. Consequently, the resistivity curve showed fluctuations during this period [37], as shown in Figure 6. This moment was defined as the air entry time *T* based on the first-order differential curve of resistivity. In the following sections, the air entry time *T* for each HVFAC mixture was determined and discussed by this method. Slowik et al. [24] also observed a sudden change in resistivity when air entered fly ash, cement mortar, and concrete. The air entry time for fly ash was 12.3 h; for cement paste, it was 2.1 h; and for concrete, it was 1.5 h.

The subsidence of coarse aggregate led to water accumulation in the matrix to form a water layer on the surface (stage I) [38]. Due to the water evaporation and self-drying of the matrix, the thickness of the water film gradually decreased until the particles on the surface were not completely covered with water. Under the action of adhesive force and surface tension, pore water among solid particles formed a meniscus, resulting in capillary pressure (stage II). With the effect of capillary pressure, pore water was forced to the surface, and surface water continued to evaporate, leading to further capillary pressure growth. When the pore water meniscus between cement particles became too small to support, air could penetrate the pore system (stage III), increasing the risk of cracking. As depicted by Figure 6, the corresponding capillary pressure threshold *P_a_* was 28.6 when the air entry time *T* was 6.7 h. Then, the pore water of the concrete matrix was rearranged, and the capillary pressure continued to increase until it reached the peak *P_max_* when air penetrated into the test area, which corresponded to the time point *T_p_* (stage IV).

As shown in Figure 6, the concrete underwent a slight initial expansion followed by rapid shrinkage in the horizontal direction, eventually flattening out. Within half an hour of pouring, the highly fluid cement slurry flowed towards both ends, causing slight expansion. During this period, there was a water film on the surface, resulting in a rapid increase in plastic shrinkage due to high evaporation rates. As a result of the ongoing process of water evaporation and cement hydration, the volume of free water in the concrete matrix decreased over time, and the strength gradually increased. As the concrete reached its final set, its shrinkage tendency became more gradual, which corresponded to the time point *T_s_* [25]. The time point at which the slope of the rapid capillary pressure development phase intersected with the initial capillary pressure is denoted as *T_q_*, representing the time capillary pressure started to increase, the slope of the capillary pressure development stage is the second slope of the segment where the peak pressure was 40–60%. The plastic shrinkage value corresponding to *T* is denoted as *ε_T_*, representing the plastic shrinkage value at the air entry time.

### 3.3. Capillary Pressure of HVFAC

#### 3.3.1. Effect of Fly Ash Content

**Figure 7:**
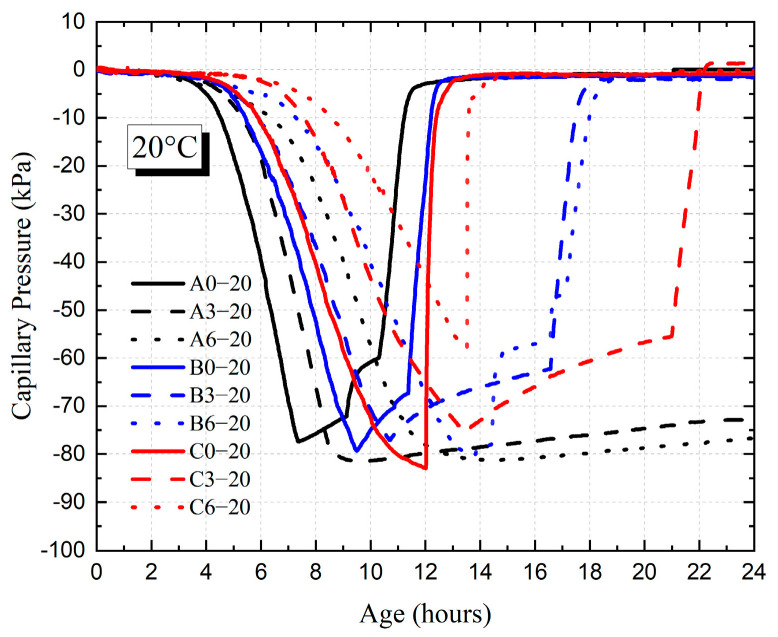
The development of HVFAC capillary pressure at 20 °C ambient temperature.

**Figure 8:**
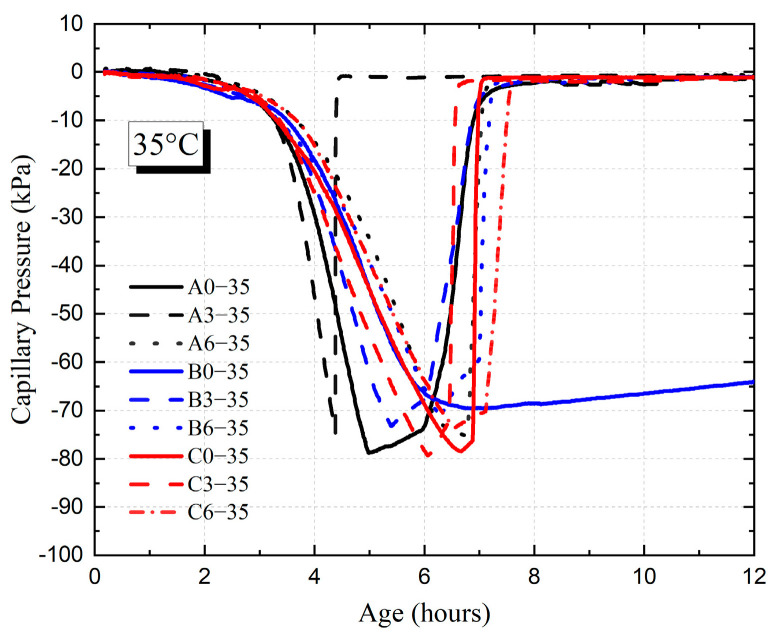
The development of HVFAC capillary pressure at 35 °C ambient temperature.

Figure 7 and Figure 8 show the development of capillary pressure for each HVFAC with different ambient temperatures. It was shown that the *P_max_* value of each HVFAC did not differ significantly at different temperatures and all of them were in the range of 70 kPa to 90 kPa. Through the above analysis method, the values of air entry time *T*, *T_q_*, and *P_a_* for each HVFAC were calculated.

**Figure 9:**
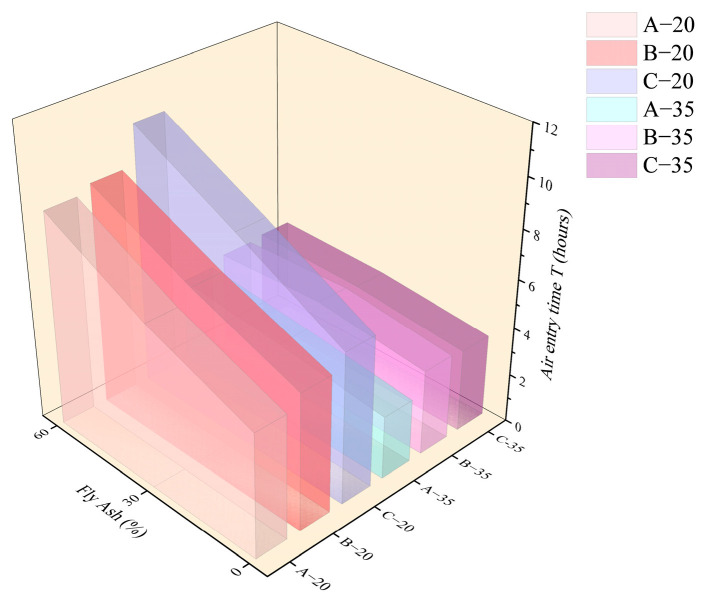
The *T* value of each group with the varying content of fly ash.

**Figure 10:**
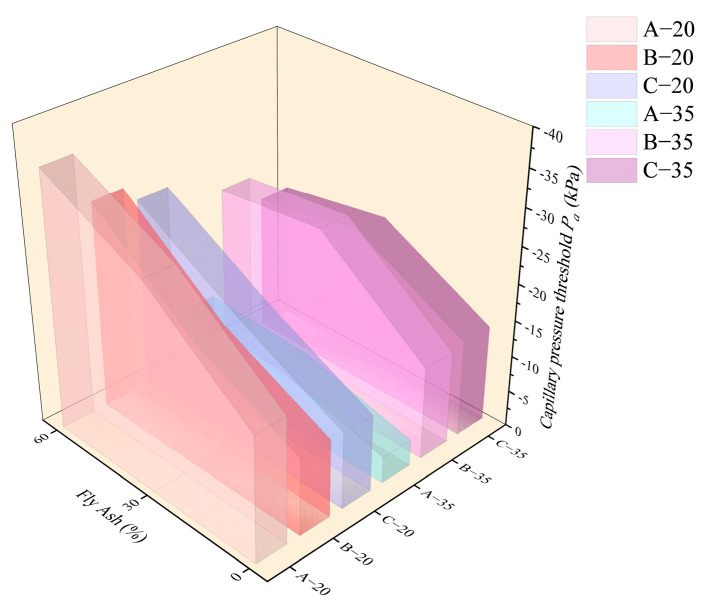
The *P_a_* value of each group with the varying content of fly ash.

Figure 9 and Figure 10 show the *T* value and *P_a_* value for each HVFAC with different temperatures. It was shown that the *T* value and *P_a_* value of each group increased with the increase in fly ash content at 20 °C. At 20 °C, compared to A0, the *T* values of A3 and A6 were delayed by 1.6 h and 3.7 h, and the value of *P_a_* was reduced by 11.1 kPa and 18.1 kPa, respectively, whereas at 35 °C, the *T* value of each group showed no significant change with the increase in fly ash content, and only slightly increased with the increase in fly ash content from 0 to 30%. The *P_a_* initially decreased and then, generally there was no significant difference between the fly ash content of 30% and 60%.

The addition of fly ash significantly decreased the threshold of concrete capillary pressure at a 20 °C environment owing to the benefit of fly ash on the size distribution and geometry of matrix pores [39]. The decrease in bigger pores and the increase in smaller pores in concrete significantly slowed down the evaporation rate of free water. Moreover, replacing the cement with the same weight of fly ash reduced the water consumed by the cement hydration reaction, which kept the water inside the concrete abundant and enabled the refilling of surface water timely, thus prolonging the existence of the water film on the surface and resulting in an increase in the *T* value and *P_a_* value.

**Figure 11:**
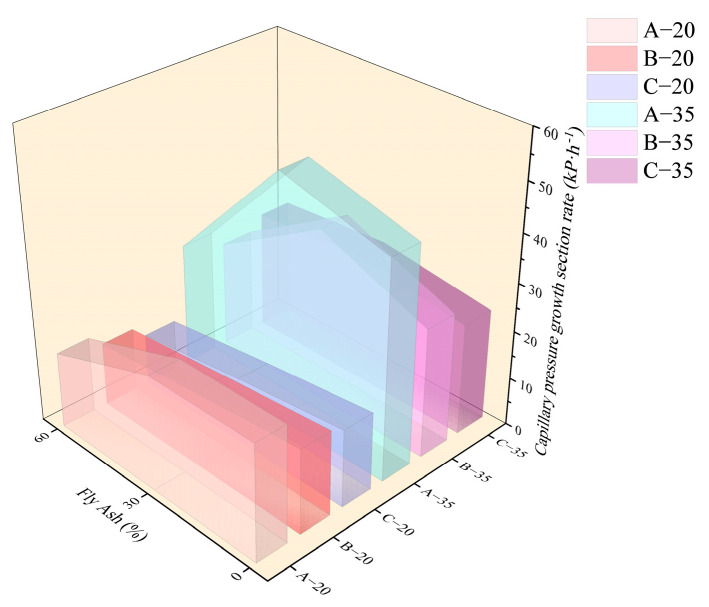
Growth period of the rapid increase in capillary pressure of each group.

**Figure 12:**
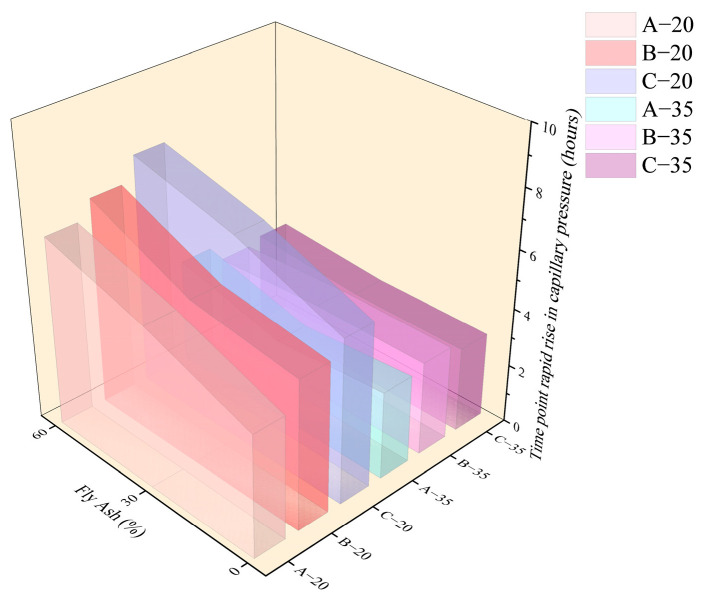
Time point of the rapid increase in capillary pressure of each group, *T_q_*.

As shown in Figure 9 and Figure 10, in a high-temperature environment, the value of *T* was advanced generally relative to the 20 °C environment. Figure 11 and Figure 12 demonstrate that the time point and growth rate of the fast increase in capillary pressure were significantly advanced and increased in the 35 °C environment compared to that of the 20 °C environment. In the 35 °C environment, the evaporation rate of water and the hydration reaction of cement were accelerated, resulting in a significant increase in free water consumption. On the other hand, although the addition of fly ash improved the pore structure, the connectivity of the pores decreased, resulting in the migration of water in the concrete matrix that cannot be transferred to the surface timely. Therefore, the difference in *T* value among all groups was minimal in a high-temperature environment, slightly increased in concrete with fly ash compared to ordinary Portland concrete. The value of *P_a_* was also insensitive to the increase in fly ash content, only increasing slightly compared to ordinary Portland concrete.

#### 3.3.2. Effect of Water–Binder Ratio

The early-age capillary pressure changes in fresh cement cementitious are relatively rapid and are significantly influenced by the water–binder ratio. Research [40] indicates that the capillary pressure of fresh cement-based materials is around 70 kPa, and it was found that the capillary pressure at the surface and bottom of the paste is the same. When the water–binder ratio decreases from 0.3 to 0.2, the time for the appearance of capillary negative pressure advances from 1 h to 0.5 h, but the water–binder ratio has a minor effect on the maximum value of capillary pressure. Other research [41] has shown that measuring the capillary pressure in early concrete reveals a maximum pressure of 80 kPa, and when the critical pressure is 20 kPa, spraying can effectively reduce capillary pressure. Slowik et al. [32] monitored the capillary pressure in early concrete and found that the corresponding capillary negative pressure when air enters is between 20 and 30 kPa, which is consistent with the test results in this paper. However, when the fly ash volume increases to 60%, the corresponding capillary negative pressure rises rapidly, indicating a greater risk of plastic cracking.

As shown in Figure 9 and Figure 10, the value of *T* increased with the increase in the water–binder ratio at 20 °C, while the value of *P_a_* decreased with the increase in the water–binder ratio first and then basically remained stable. At 35 °C, the value of *T* for group A decreased compared to groups B and C, and there was almost no difference between groups B and C, while the value of *P_a_* increased with the increase in the water–binder ratio first and then basically remained stable. At 20 °C, the HVFAC with a high water–binder ratio contained a larger amount of free water, and the water on the concrete surface can be replenished timely after evaporation. The water film on the surface persisted for a longer time, which meant that it took more time for air to break through the water film and enter the cement matrix, resulting in an increased *T* value. The water–binder ratio increased, causing the formation of wider and better-connected capillary networks in the cement matrix, thus slowing down the development of capillary pressure [36]. However, the effect of the water–binder ratio on capillary pressure was limited, and consequently, *P_a_* decreased first and then remained almost unchanged with the increase in the water–binder ratio.

At 35 °C, the value of air entry time *T* was significantly decreased in each group compared to that at 20 °C environment. Especially for concrete with low water–binder ratio (group A), the internal free water was rapidly consumed by the hydration reaction, which made the connectivity of concrete internal pores poor and caused the water on the concrete surface not to be replenished timely; hence, the values of *T* and *P_a_* were small. However, in a high-temperature environment, there was no significant effect of increasing the water–binder ratio on delaying the *T* value, because the rate of water evaporation was the major factor affecting the *T* value at high-temperature conditions.

### 3.4. Plastic Shrinkage of HVFAC

#### 3.4.1. Influence of Fly Ash Content

The research [2] indicates that for the early plastic shrinkage of mortar with 030% fly ash replacement, the plastic shrinkage significantly decreases at a 20% replacement level, and further increasing the fly ash content results in very little change in shrinkage values. Wang et al. [42] investigated the shrinkage of concrete in the amount of 0–40% admixture. The results showed that the addition of fly ash improved the resistance of the concrete to plastic shrinkage but also increased the risk of drying-induced cracking under confinement. Wang et al. [43] investigated the effect of different admixtures of fly ash on the plastic shrinkage cracking of fiber concrete, and the results showed that the addition of fly ash affects the pore structure and plastic shrinkage behavior of the paste.

**Figure 13:**
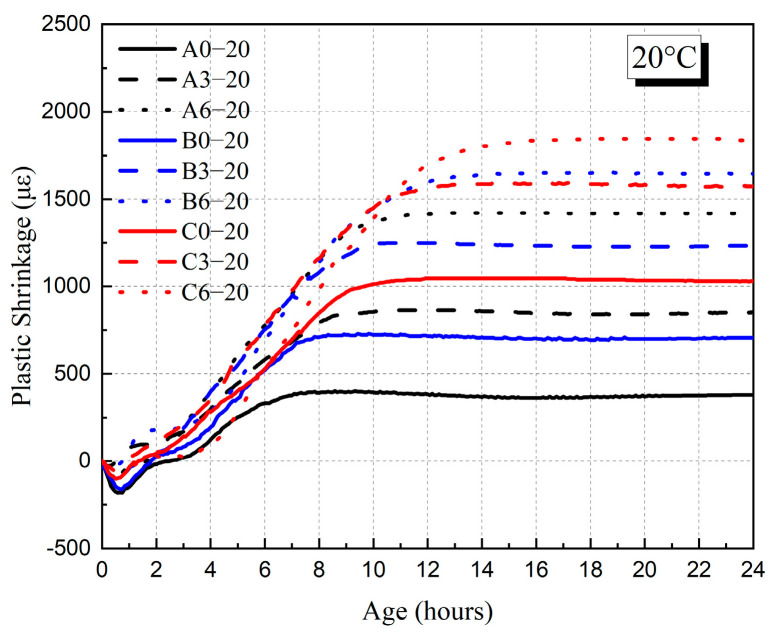
The plastic shrinkage of HVFAC before 24 h under 20 °C environment.

**Figure 14:**
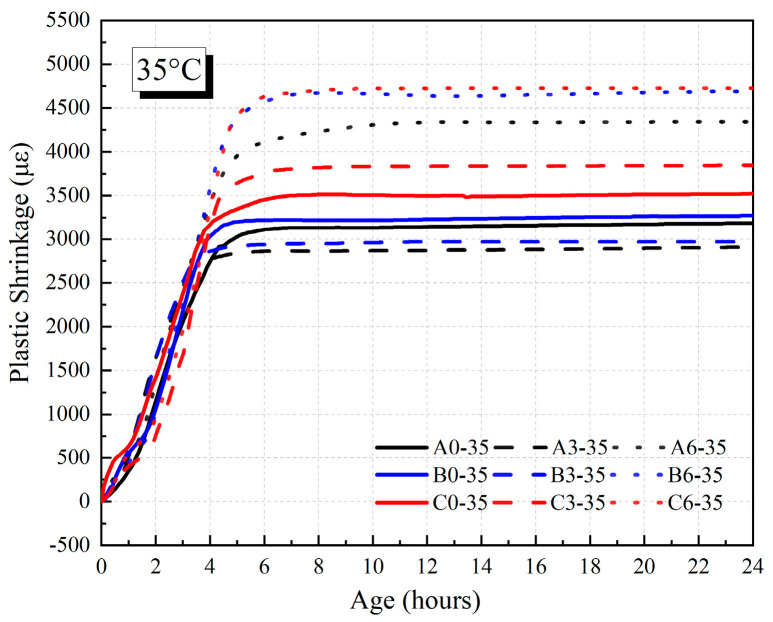
The plastic shrinkage of HVFAC before 24 h under 35 °C environment.

**Figure 15:**
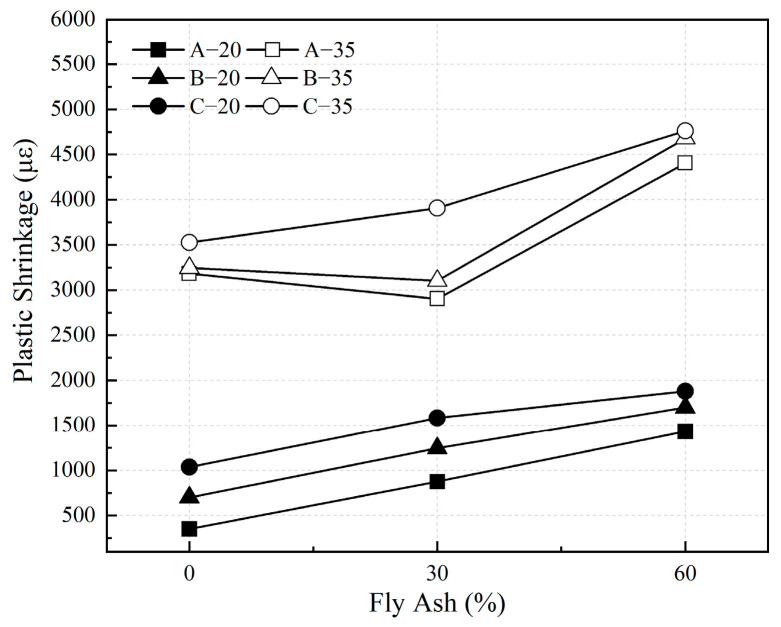
The plastic shrinkage of HVFAC at 24 h under different environments.

The development of plastic shrinkage of HVFAC before 24 h in 20 °C and 35 °C environments are shown in Figure 13 and Figure 14. The values of plastic shrinkage of the HVFAC at 24 h are shown in Figure 15. The results indicate that the plastic shrinkage of HVFAC increased with the increase in fly ash content in 20 °C environment conditions. Moreover, the nucleation effect of fly ash accelerated cement hydration while its own reaction was relatively slow. The fly ash products filled the already-formed pores, thereby refining the pore structure [44]. This optimized the concrete’s pore structure and decreased the rate of water evaporation, thus the time of reaching the peak of negative capillary pressure was delayed, and the time of plastic shrinkage into the smoothing phase was also delayed accordingly. Drying shrinkage is generally considered the dominant form of shrinkage for the water–binder ratio of concrete exceeding 0.3 [45]. Especially for the concrete with fly ash, the internal moisture was more abundant, which resulted in the water evaporating from the surface being greater, so early-age drying shrinkage was relatively large [46].

At 35 °C, the plastic shrinkage of concrete with 0.4 and 0.5 water–binder ratios first slightly decreased and then increased with the increase in fly ash content. For concrete with a high water–binder ratio (group C), the plastic shrinkage increased with the increase in fly ash content. In the high-temperature environment, the shrinkage caused by water evaporation was dominant, so the development of negative capillary pressure and plastic shrinkage were not significantly different in each group. On the one hand, the addition of fly ash improved the pore structure of concrete and reduced the rate of water evaporation, which contributed to the reduction in plastic shrinkage. On the other hand, the water consumed in the hydration reaction at the early stage was decreased with the increasing content of fly ash, and the amount of water evaporated from the unit volume of concrete was increased. Under the combination effect of both, the plastic shrinkage value of concrete with 30% fly ash content was lower. For the concrete with high water–binder (group C), the latter dominated the development of plastic shrinkage, so the plastic shrinkage increased with the increase in fly ash content.

#### 3.4.2. Influences of Water–Binder Ratio and Curing Temperature

Research [47] showed that the higher the water–binder ratio, the higher the early plastic shrinkage at different cement finenesses. Nasir et al. [48] investigated the plastic shrinkage of concrete at water–binder ratios ranging from 0.3 to 0.45, and the results showed that the higher the water–binder ratio, the higher the early plastic shrinkage.

It has been shown that the curing temperature has a large effect on plastic shrinkage, and it is now generally accepted that 35 °C is the maximum mixing temperature in hot weather. Meyer et al. [31] investigated the plastic shrinkage of concrete at curing temperatures of 25–35 °C and found that an increase in both ambient and initial concrete temperatures resulted in an increase in the shrinkage of concrete in the plastic state. Nasir [49] investigated the relationship between casting temperature (25 °C, 32 °C, 38 °C, and 45 °C) and the plastic shrinkage of concrete under high-temperature conditions (35 °C) and showed that the casting temperature had the greatest effect on the plastic strain, with high and lowest plastic shrinkage strains measured for both 25 °C and 30 °C degree specimens at different admixtures and curing methods, respectively.

Figure 13 and Figure 14 illustrate that the plastic shrinkage deformation of concrete increased significantly with the increase in the water–binder ratio at 20 °C and 35 °C. With the increase in the water–binder ratio, the free water in the concrete increased, and more water was evaporated in drying condition, resulting in an increase in plastic shrinkage. In addition, the water loss led to an increase in capillary pressure, which further contributed to an increase in the plastic shrinkage of concrete. The plastic shrinkage of concrete increased in all the groups as the curing temperature increased. This was mainly due to the acceleration of the water evaporation rate and the sharp increase in capillary pressure as the curing temperature increased. As shown in Figure 14 and Figure 15, the growth rate and value of plastic shrinkage increased significantly under the high-temperature environment.

## 4. Discussion

**Figure 16:**
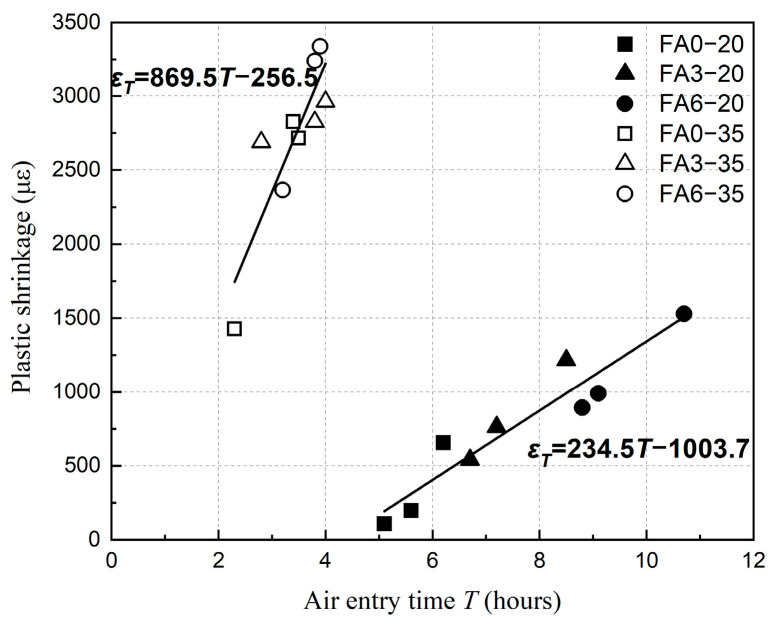
Relationship between air entry time *T* and *ε_T_* at different ambient temperatures.

Figure 16 shows the relationship between the air entry time *T* and *ε_T_* (the value of plastic shrinkage at time point *T*). It shows a well-linear relationship between *T* and *ε_T_* at both 20 °C and 35 °C environments. The law indicates that the plastic shrinkage is mainly dependent on the rate of water evaporation before time point *T*, and is proportional to the length of time the concrete was exposed to the environment. Compared with the 20 °C environment, the development of plastic shrinkage was significantly faster in the 35 °C environment, which was mainly due to the increased rate of water evaporation at high-temperature conditions.

For the concrete with high fly ash content, the impact of the high-temperature environment on air entry time *T* was more obvious than the plastic shrinkage compared with the ordinary Portland concrete. With regard to the concrete of groups A, B, and C, the value of air entry time *T* of ordinary Portland concrete decreased by 55%, 38%, and 45% and plastic shrinkage increased by 1209%, 1271%, and 330%, respectively, owing to the elevated of ambient temperature, while the value of air entry time *T* of concrete with 60% content fly ash decreased by 64%, 58%, and 64% and plastic shrinkage increased by 164%, 227%, and 118%, respectively. It was because the values of *T* and plastic shrinkage were primarily determined by the content of fly ash in the 20 °C environment. However, in the high-temperature environment, the rate of water evaporation was th e key factor affecting these parameters, which resulted in a small difference in the air entry time *T* and the plastic shrinkage of each concrete.

**Figure 17:**
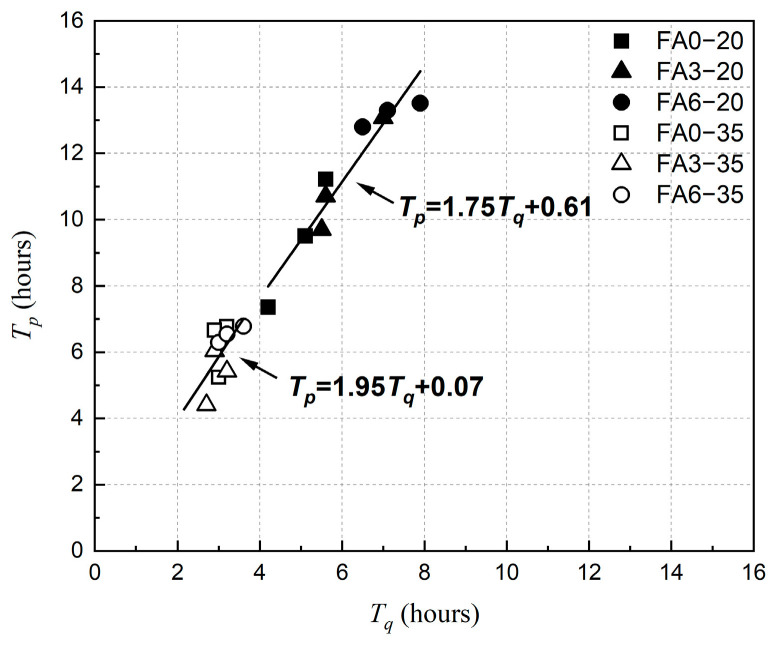
Relationship between *T_q_* and *T_p_* at different ambient temperatures.

**Figure 18:**
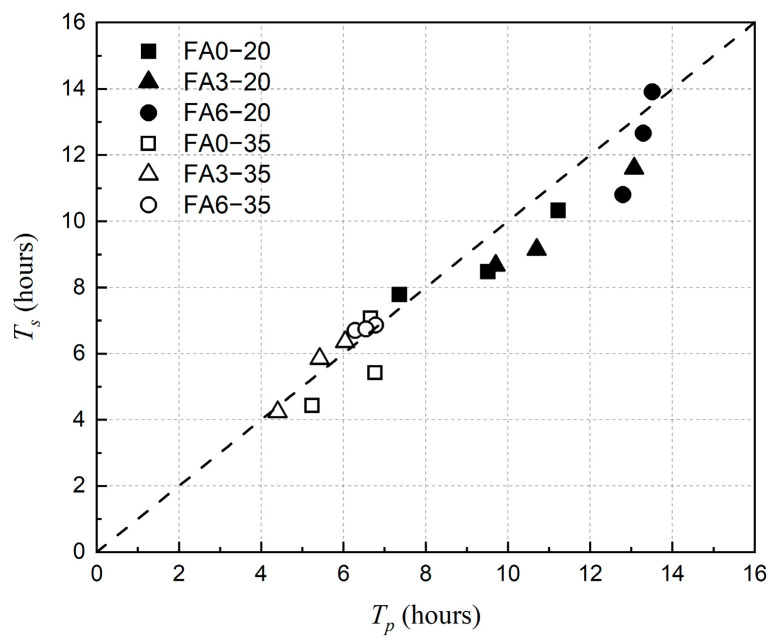
Relationship between *T_p_* and *T_s_* at different ambient temperatures.

Figure 17 and Figure 18 show the relationship between the *T_p_* (the time point that the *P_max_* was developed) with *T_q_* (the time point that capillary pressure started to rise) and *T_s_* (the time point that plastic shrinkage stabilized). As shown in Figure 17, it also showed a good linear relationship between *T_q_* and *T_p_* at both 20 °C and 35 °C environments. The linear relationship between *T_q_* and *T_p_* was more pronounced in the 20 °C environment. Due to the faster curing process of concrete in the 35 °C environment, the discrepancy of *T_q_* and *T_p_* was not significant for each concrete, with the value of *T_p_* being approximately twice as high as the value of *T_q_*.

Figure 18 showed that there was a well corresponding relationship between *T_p_* and *T_s_*. It reflected that the point at which the capillary pressure was maximal corresponding with the plastic shrinkage entered the stabilized stage, which also meant the internal skeleton of concrete was basically formed. Therefore, it was possible to timely obtain the curing state of concrete through the variation in capillary pressure, which will provide guidance for the early-age cracking resistance of concrete.

Fly ash has an annual global production of approximately 1.2 billion tons. Conventional disposal methods (landfilling or stockpiling) not only occupy substantial land resources but also pose contamination risks to soil and water systems. The incorporation of high-volume fly ash in concrete production demonstrates significant potential for carbon emission reduction, primarily attributed to partially substituting cement, thereby diminishing CO_2_ emissions from cement manufacturing processes [50]. As the proportion of fly ash increases, the carbon footprint associated with concrete production can be substantially mitigated. In 40 MPa grade concrete design formulations, a 25% fly ash replacement ratio achieves 13–15% carbon reduction [51]. Further increasing the fly ash content (30–80%) enables more pronounced emission reductions. However, HVFAC requires optimized mix proportions, enhanced curing regimes, and potential integration with other low-carbon technologies to balance mechanical performance and durability requirements [52].

## 5. Conclusions

In this paper, the effects of fly ash admixture, water–binder ratio, and ambient temperature on the air entry time *T*, capillary pressure, and plastic shrinkage of HVFAC were investigated by designing nine different fly ash concrete materials. The following conclusions were obtained:

The present study investigates the air entry time *T* of HVFAC under various curing temperatures. The results demonstrate that *T* increases with an increase in the water–binder ratio and fly ash admixture at 20 °C, while the effect of *T* is negligible at 35 °C. Moreover, *T* increases with an increase in the curing temperature. However, the addition of fly ash admixture and an increase in the water–binder ratio do not significantly affect the *T* value at 35 °C.

The capillary pressure threshold *P_a_* of HVFAC varies with the water–binder ratio and fly ash admixture under different curing temperatures. At 20 °C, *P_a_* decreases with an increase in fly ash admixture and increases first and then stabilizes with an increase in the water–binder ratio. At 35 °C, *P_a_* first decreases and then increases with an increase in fly ash admixture, and first decreases and then stabilizes with an increase in the water–binder ratio. At low water–binder ratios, *P_a_* increases with increasing curing temperature, while at high water–binder ratios, there is no significant trend observed for *P_a_*.

The plastic shrinkage of HVFAC increases significantly with the increase in curing temperature. At 20 °C, the increase in fly ash admixture and water–binder ratio leads to the increase in plastic shrinkage of HVFAC in the early stage. At 35 °C, the plastic shrinkage increases with the increase in water–binder ratio, decreases first and then increases with the increase in fly ash admixture, and 30% fly ash admixture has the minimum plastic shrinkage. There is a linear correlation between the air entry time *T* and the plastic shrinkage value at this time, and the latter increases with the increase in the former. The larger the *T* value is, the higher the crack resistance is.

## Figures and Tables

**Table 1:** Particle grading of fine aggregate.

Sieve Aperture (mm)	Sieve Residue (g)	Separate Sieve (%)	Cumulative Sieve (%)
4.75	11	2.2	2.2
2.36	31	6.3	8.5
1.18	56	11.2	19.7
0.6	146	29.3	49.0
0.3	214	43.0	92
0.15	26	5.2	97.2

**Table 2:** Particle grading of crushed coarse aggregate.

Sieve Aperture (mm)	Sieve Residue (g)	Separate SIEVE (%)	Cumulative Sieve (%)
9.5	775	22.4	22.4
4.75	2516	72.9	95.3
2.36	151	4.4	99.7

**Table 3:** Mix proportion and strength of concrete.

Series	No.	FA (%)	W/B	Unit Mass (kg/m^3^)						*f_c_* (MPa)
				Cement	FA	Water	Sand	Gravel	SP	
A	A0	0	0.4	450	0	180	654	1113	0.675	70.8
	A3	30		310	133	177	634	1096	0.576	51.2
	A6	60		174	262	174	634	1078	0.828	26.6
B	B0	0	0.5	360	0	180	698	1139	0.684	58.5
	B3	30		249	107	178	689	1125	0.640	35.7
	B6	60		140	211	175	681	1110	0.684	17.4
C	C0	0	0.6	300	0	180	736	1152	0.600	41.9
	C3	30		208	89	1178	728	1140	0.564	25.9
	C6	60		1117	176	176	721	1128	0.573	12.8

Note: FA represents fly ash, W/B represents water–binder ratio, *f_c_* represents compressive strength at 28 d, and SP represents superplasticizer; contents of SP are based on the total mass of cementitious materials.

**Table 4:** Chemical compositions of cement and fly ash (wt.%).

Material	SiO_2_	Al_2_O_3_	Fe_2_O_3_	CaO	MgO	SO_3_	Others
Cement	22.6	5.04	3.33	61.57	2.65	2.31	2.50
Fly ash	42.77	30.28	6.08	14.00	2.26	0.81	3.80

## Data Availability

The original contributions presented in this study are included in the article. Further inquiries can be directed to the corresponding author.
